# Spatiotemporal disparities in maternal mortality and the role of multiscale administrative levels: a 20-year study across Chinese counties

**DOI:** 10.3389/fpubh.2025.1572382

**Published:** 2025-05-09

**Authors:** Lingfeng Liao, Fengling Yuan, Yaqian He, Shixi Xu, Xingyi Tang, Mingyu Xie, Xianteng Tang, Zhangying Tang, Guo Zeng, Yumeng Zhang, Chao Song

**Affiliations:** ^1^HEOA-West China Health & Medical Geography Group, West China School of Public Health and West China Fourth Hospital, Sichuan University, Chengdu, Sichuan, China; ^2^Institute for Healthy Cities and West China Research Centre for Rural Health Development, Sichuan University, Chengdu, Sichuan, China; ^3^Department of Geosciences, University of Arkansas, Fayetteville, AR, United States; ^4^School of Population Medicine and Public Health, Chinese Academy of Medical Sciences and Peking Union Medical College, Beijing, China; ^5^School of Public Health, Xi'an Jiaotong University, Xi'an, Shaanxi, China; ^6^State Key Laboratory of Oil and Gas Reservoir Geology and Exploitation, School of Geoscience and Technology, Southwest Petroleum University, Chengdu, China

**Keywords:** MMR, small area, spatiotemporal heterogeneity, multiscale effects, spatial inequity lock-in effect, SDG 3, county level, China

## Abstract

**Background:**

China has made progress in reducing maternal mortality ratio (MMR), yet county-level spatiotemporal heterogeneity persists. This study aims to identify spatiotemporal disparities in MMR and quantify the impacts of various administrative levels on these disparities.

**Methods:**

We analyzed county-level MMR panel data from 1996 to 2015, employing the spatial Gini coefficient, Anselin Local Moran's I, and Getis-Ord Gi^*^ to assess spatiotemporal disparities related to spatial inequity and geographic clustering. Additionally, we applied a Bayesian multiscale spatiotemporally varying intercepts (BMSTVI) model to unveil the national temporal trend and multiple sub-national spatial patterns in maternal mortality risk. We further quantified the relative contributions of five sub-national administrative levels using the spatiotemporal variance partitioning index (STVPI).

**Results:**

Results suggested that from 1996 to 2015, the proportion of MMR in counties achieving Sustainable Development Goals (SDGs) increased from 27.05% to 93.40%, yet spatiotemporal disparities remained. The spatial Gini coefficient and geographic clustering analyses indicated temporally varying but spatially stable inequities patterns, highlighting the Spatial Inequity Lock-in (SILI) effect. Hotspot analysis identified sensitive and exemplary counties, underscoring the need for targeted regional interventions. The BMSTVI model indicated a declining trend in MMR risk over 20 years, with the most substantial reduction from 2003 to 2012. While the geographic distribution of high-risk areas remained relatively stable, analyses at finer administrative levels enabled more precise identification of affected locations and improved intervention effectiveness. Finally, the STVPI revealed that spatial effects contributed 83.91% (95% CIs: 78.66%−89.47%) to MMR variations, far exceeding the 11.60% (95% CIs: 7.27%−16.55%) from temporal effects. The contribution from the administrative county-level was the highest (29.15%, 95% CIs: 19.69%−35.06%), followed by contributions from the seven geographical regions (14.10%, 95% CIs: 6.61%−34.06%), rural–urban differences (13.77%, 95% CIs: 4.93%−39.2%), provincial level (12.41%, 95% CIs: 8.06%−16.85%), and city level (11.21%, 95% CIs: 7.53%−13.84%).

**Discussion:**

These findings underscore the crucial need for region-specific, time-sensitive policies to achieve maternal health equity across Chinese counties. This study provides a robust empirical foundation for a multi-tiered adaptive policy framework grounded in systematic spatiotemporal assessment across macro, meso, and micro scales to guide targeted maternal health interventions globally.

## 1 Introduction

Improving maternal health is a global health priority, as it is crucial for the well-being of pregnant women and their families, serves as a key indicator of social and economic progress, and reflects advancements in healthcare, equity, and overall societal well-being (1). According to the World Health Organization's (WHO) definition, maternal mortality refers to the death of a woman during pregnancy or within 42 days of its termination. Maternal mortality ratio (MMR) is defined as the number of maternal deaths per 100,000 live births, following the WHO standard calculation (2). MMR, a key health indicator for women of reproductive age and vulnerable groups, not only reflects the level of national healthcare (3), but also plays an indispensable role in assessing health equity at national and sub-national level (4) and in guiding health policy formulation and implementation (5).

The global MMR declined substantially during the Millennium Development Goals (MDGs) period, which sought to achieve a three-quarters reduction in MMR from the 1990 value by 2015 (6). However, despite these improvements, many countries and territories have yet to achieve the Sustainable Development Goals (SDGs) target of reducing MMR to <70 per 100,000 live births (4). In addition, pronounced MMR inequities persist both among countries and at the sub-national level (e.g., economically diverse regions, states or provinces, cities, and counties) of the same country (6).

In some countries that have achieved the SDGs, national-level achievements can obscure significant sub-national inequities in maternal health, especially within small areas. Unfortunately, previous studies have predominantly focused on larger administrative scales (e.g., states, provinces), neglecting granular analyses at smaller administrative units, such as counties (7). Large-scale spatial assessments typically overlook critical aspects of small-area geospatial disparities, particularly spatial inequity and geographic clustering (8). Consequently, one-size-fits-all policies fail to address specific local needs (9), further exacerbating geospatial disparities in MMR (10).

Spatial inequity assessment, grounded in the first law of geography, surpasses traditional non-spatial methods by more precisely capturing intra-regional disparities and small-area variations through considering spatial autocorrelation (11). Additionally, geographic clustering phenomena reveal the regional Matthew effect in health outcomes: medically privileged regions, supported by economic and policy advantages, attract more healthcare resources and medical professionals, creating a positive feedback loop; in contrast, medically underserved regions risk falling into a negative feedback cycle (3, 4, 8). Such geographic clustering polarization exacerbates regional inequities, an aspect often overlooked in previous MMR equity evaluations (1). Moreover, limitations of prior MMR assessments stem from the separation of spatial and temporal dimensions (2), inadequately capturing the complexity of sub-national disparities (12). Spatiotemporal coupling analysis, integrating both spatial and temporal dimensions, offers a more comprehensive framework for understanding and addressing disparities related to spatial inequity and geographic clustering in maternal health, thus enabling targeted and effective maternal health policy interventions (13).

Building upon the identification of spatiotemporal inequities and clusters described above, it is imperative to investigate the underlying drivers contributing to maternal mortality disparities. Due to substantial differences in resource allocation and policy implementation across multiple administrative levels, administrative scale can significantly influence heterogeneous maternal health outcomes, particularly at smaller spatial scales (14). Hence, systematically quantifying disparities in MMR across different administrative levels can more accurately assess the effectiveness and impact of health policies implemented at each administrative level (15). Although some studies have considered the scale effects in health outcomes (16), research specifically addressing administrative scale effects on MMR remains limited, especially within the context of spatiotemporal coupling involving multiple administrative levels (17). Addressing this gap is critical to fully understanding the complex, multidimensional nature of maternal health disparities.

In China, examining the spatiotemporal disparities of MMR and the potential influence of administrative scale on these disparities is critically important, yet remains largely unexplored. First, despite significant national advancements in maternal health across China in recent years, pronounced spatiotemporal inequities in MMR persist, particularly at smaller geographic scales (18). Second, China's multi-tiered administrative structure uniquely supports MMR reduction by integrating top-down policy directives with bottom-up adaptability through hierarchical governance. Specifically, the five-tier system, namely, central, provincial, city, county, and grassroots levels, enables strategic policy formulation at the macro level, where the central government sets national targets, enforces maternal healthcare legislation, and allocates fiscal resources to underdeveloped regions (19–21). At the meso level, provincial and city governments contextualize these national strategies through local innovations, such as the establishment of three-tier maternal-child healthcare networks, deployment of digital health platforms for real-time risk monitoring, and formation of medical alliances to bridge rural–urban service gaps (22). At the micro level, county and grassroots authorities prioritize equitable service delivery by standardizing township obstetric units, assigning village doctors for prenatal home visits, and implementing poverty-alleviation measures, such as the “Two Exemptions and One Subsidy” policy, to reduce healthcare costs for disadvantaged families (23–25). Consequently, China's vertically integrated, multiscale governance framework has facilitated coordinated and synergistic progress in maternal health outcomes nationwide (26, 27).

To address above issues, this research uses China as a case study, leveraging MMR data from 2,894 counties between 1996 and 2015, systematically analyzing these spatiotemporal disparities and examining the critical role of multiscale administrative levels. The objectives of this research are structured as follows. First, we examine disparities in MMR by addressing two key aspects: spatiotemporal inequities and spatiotemporal clusters. Second, we assess the contributions of multiscale administrative levels, spanning rural–urban differences, seven geographical region, and administrative levels including provincial level, city level, and county level, to MMR disparities. By establishing a robust empirical framework, this study delivers actionable insights for designing region-specific and time-sensitive maternal health policies, thereby advancing SDGs through targeted small-area interventions.

## 2 Materials and methods

### 2.1 Study area and data

**Figure 1:**
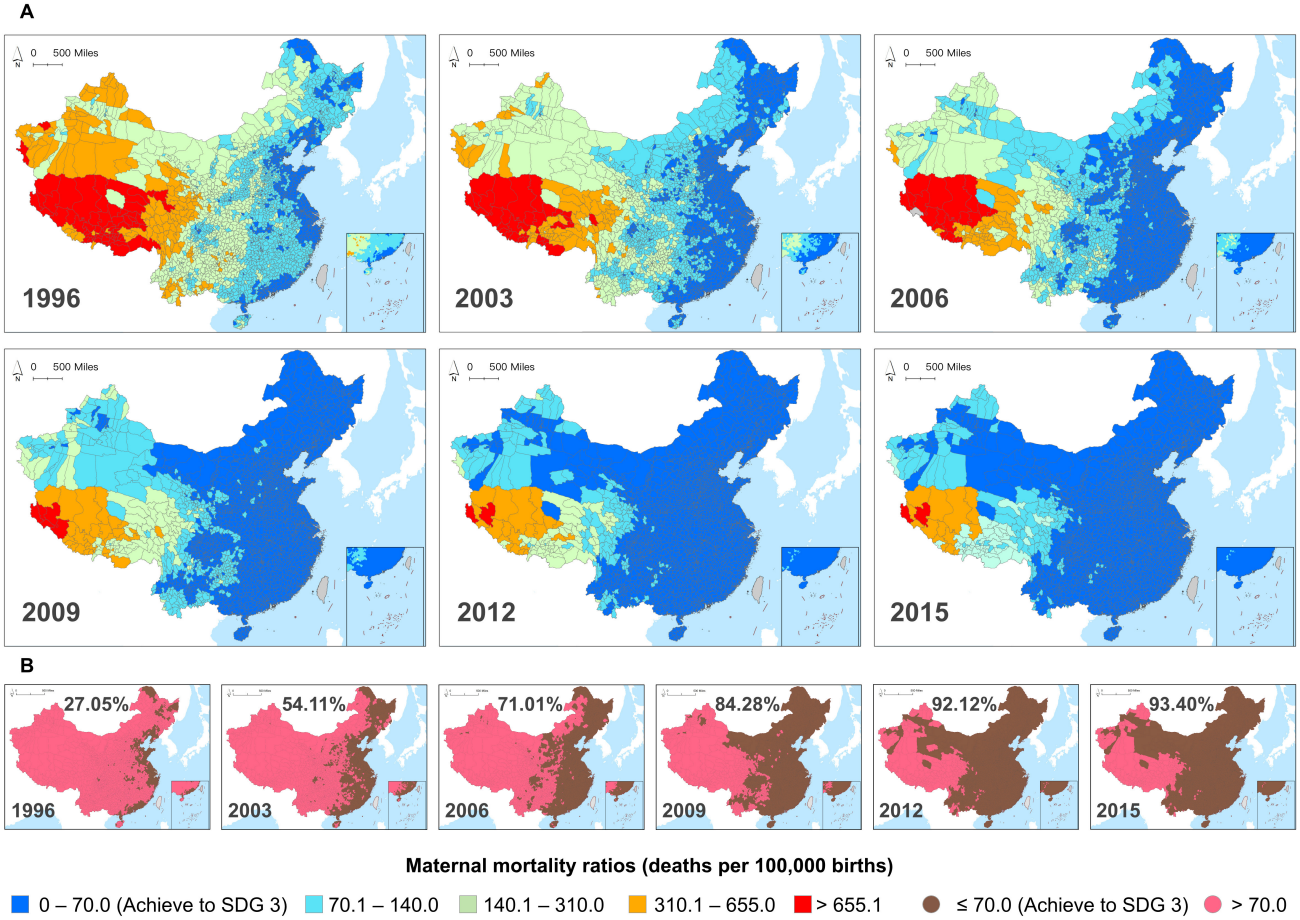
**(A)** County-level maternal mortality ratios (MMR) in Chinese mainland from 1996 to 2015. **(B)** The binary classification maps display counties that have achieved SDG 3 by reducing MMR to below 70 per 100,000 live births, and counties that have not achieved this.

We collected the MMR panel data from 1996 to 2015 at the Chinese county level. These data are sourced from surveys, monitoring systems, censuses, and the Annual Report on Maternal and Child Health System (ARMCH) (28). Using ArcGIS software, we generated spatial distribution maps of MMR across 2,894 counties in China. Figure 1A displays the spatial distribution of MMR in selected years, reflecting China's overall progress toward achieving SDGs targets related to MMR. Figure 1B shows in 1996, only 783 counties (27.05%) achieved SDG 3 (the target of reducing MMR to <70 per 100,000 live births by 2030), primarily concentrated in coastal regions such as Heilongjiang, Shandong, Shanghai, and Guangdong provinces. By 2015, 2,703 counties (93.4%) in China had achieved this goal. The remaining 191 counties (6.6%) not yet achieving SDG 3 are mainly distributed in Tibet, Xinjiang, and Qinghai provinces, with most of these regions being on the verge of reaching the target. To systematically evaluate variations across different administrative levels, we categorize MMR disparities into macro (national, rural–urban, and seven geographic regions), meso (provincial and city), and micro (county) levels, following established public health and health policy frameworks rather than ecological or biological classifications.

### 2.2 Research design

**Figure 2:**
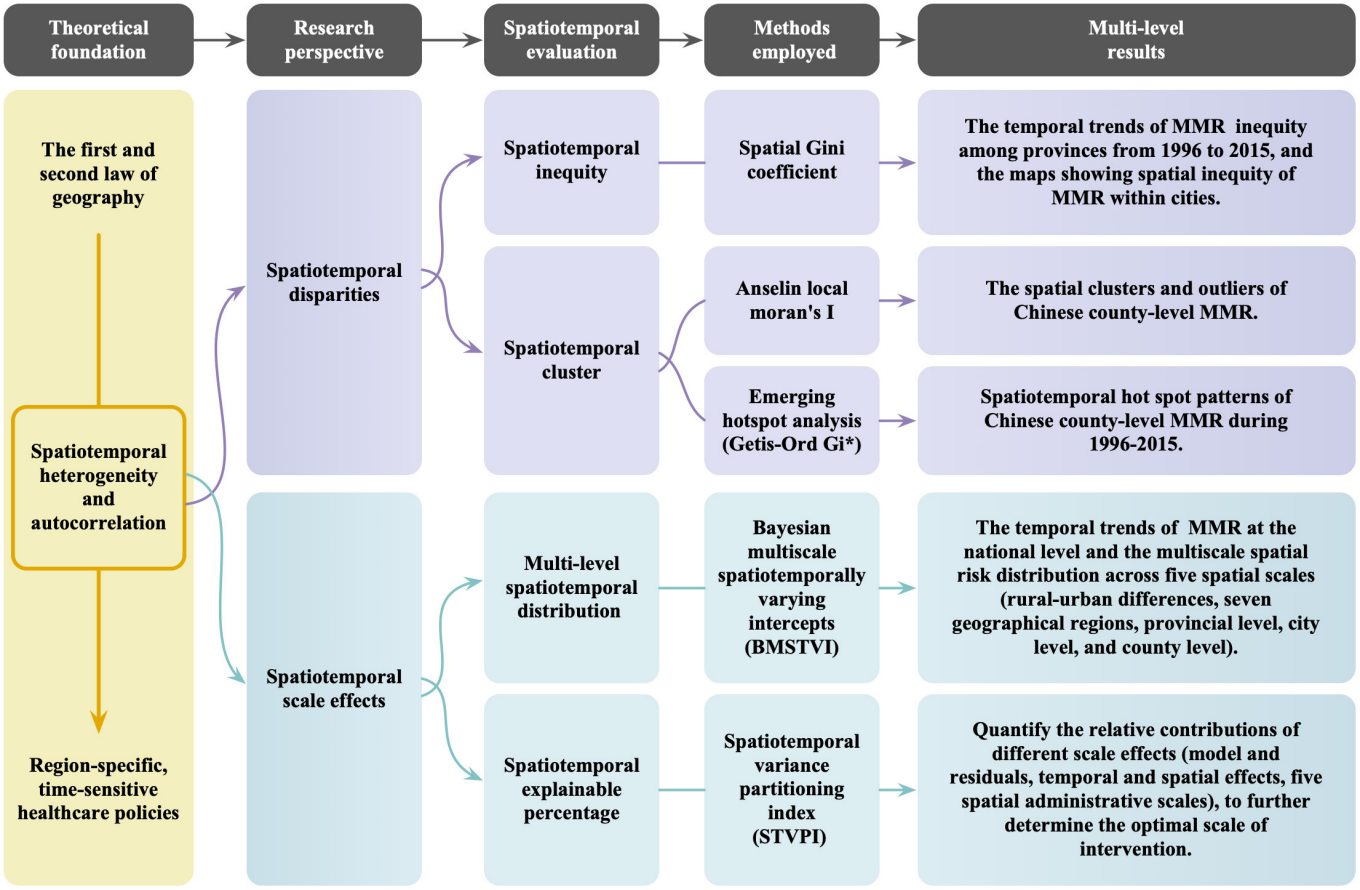
Flowchart for assessing spatiotemporal disparities (spatial inequity and geographic clustering) and the role of multiscale administrative levels: using Chinese county-level maternal mortality ratios (MMR) as an example.

Our study is grounded in the principles of spatiotemporal autocorrelation (the first law of geography) and spatiotemporal heterogeneity (the second law of geography). We investigate the multifaceted nature of small-area MMR through the lenses of spatiotemporal disparities and spatiotemporal scale effects, focusing on aspects including spatiotemporal inequity, geographic clustering, multi-scale distribution, and spatiotemporal contributions, as illustrated in Figure 2.

Specifically, the spatiotemporal inequity assessment reveals both the trends in MMR at the provincial level and the spatial distribution patterns at the city level. The spatiotemporal clustering analysis reveals anomalous regions with significant differences from surrounding areas, highlighting small areas where MMR either consistently remained high or risen over time, thus identifying areas in need of focused intervention. Furthermore, by analyzing MMR at different administrative scales and the national-level trends in MMR, we reveal the spatiotemporal heterogeneity in MMR. Quantifying the contributions of various administrative scales allows us to determine the optimal intervention level. These findings offer region-specific, time-sensitive evidence essential for optimizing policies aimed at reducing small-area MMR disparities and promoting greater equity in maternal health outcomes.

To address these evaluation dimensions, we employed advanced geospatial statistical methods to analyze county-level MMR data from China as a case study. First, we used the spatial Gini coefficient to dynamically assess the annual spatiotemporal inequities in MMR. Second, we applied Local Moran's *I* and emerging spatiotemporal hotspot analysis to detect geographic clustering patterns, incorporating temporal variations to identify both exemplary and sensitive counties. Third, we applied the Bayesian multiscale spatiotemporally varying intercepts (BMSTVI) model and the spatiotemporal variance partitioning index (STVPI) method to reveal the spatiotemporal heterogeneity of MMR across administrative scales and quantify their relative contributions. The following sections provide a detailed introduction of the implementation of these methods within our spatiotemporal evaluation framework.

#### 2.2.1 Spatiotemporal inequity assessment

The spatial Gini coefficient is a key tool for assessing spatial inequity distribution (29), accounting for spatial autocorrelation effects (30). Unlike the traditional Gini coefficient, it can evaluate small-area MMR inequities by incorporating both neighboring and non-neighboring components (19, 21, 22). The neighboring component reflects the influence of adjacent regions, while the non-neighboring component focuses on inequity within the target region itself. Values range from 0 to 1, with higher values indicating greater inequity (31). To accurately reflect MMR inequity levels and distributions, we divided the spatial Gini coefficient range (0–0.33) into five intervals: 0–0.07, 0.07–0.14, 0.14–0.21, 0.21–0.28, and 0.28–0.32 and assigned colors accordingly. Using “lctool” package (32) in *R* 4.3.3 with a neighborhood level of 2, we calculated the spatial Gini coefficient for county-level MMR across provinces and cities from 1996 to 2015, mapping the dynamic evolution of spatial inequity at the city level using ArcGIS Desktop.

#### 2.2.2 Spatiotemporal cluster assessment

Calculating the spatial Gini coefficient alone is insufficient to comprehensively characterize spatial disparities in MMR. To further explore inequities, we applied Anselin Local Moran's *I* in ArcGIS (33–35), which can adjust multiple spatial weight matrices to identify clusters of high and low values as well as spatial outliers. This method allows us to capture local spatial autocorrelation characteristics in county-level MMR and reveal geographic clusters and anomalies. A positive *I*-value with a positive *Z-*value or a negative *I*-value with a negative *Z-*value indicates that a county forms a similar high or low MMR cluster with its neighboring counties. Conversely, counties with opposite *I-* and *Z*-values are marked as spatial outliers, indicating they have an inverse relationship with neighboring counties. The results classify county-level MMR into five types: high-high clusters, low-low clusters, high-low outliers, low-high outliers, and counties with no statistically significant patterns.

Since Local Moran's *I* only focus on clustering at specific time points, we used ArcGIS Pro to create a space-time cube and conducted a spatiotemporal hotspot analysis (Getis-Ord Gi^*^) (25, 26) to further explore spatiotemporal coupling trends of MMR at the county level in China from 1996 to 2015. When the clustering intensity of MMR surpasses the predefined threshold, it generates a hotspot, while values below this threshold indicate the presence of a cold spot. Positive indices highlight areas with high-value clusters, while negative indices identify areas with low-value clusters. This analysis identifies 17 patterns, including diminishing hotspots, historical hotspots, oscillating cold spots, intensifying cold spots, persistent cold spots, and new cold spots, providing a dynamic depiction of spatiotemporal clusters in MMR in China.

#### 2.2.3 Spatiotemporal scale effects of different administrative levels

We employed two advanced methodologies to examine the relative contributions of five administrative divisions to MMR disparities, i.e., rural–urban differences, seven geographical regions, 31 provinces, 360 cities, and 2,894 counties. First, we developed a Bayesian multiscale spatiotemporally varying intercepts (BMSTVI) model to detect the overall national temporal heterogeneous trend and multilevel spatial heterogeneous distributions of MMR (36). Using “BSTVC” package in *R* 4.3.3 (37), we captured spatiotemporal variations in MMR, and outputted temporal risk trends nationwide from 1996 to 2015 and spatial distribution maps of average risk for five administrative scales. Equations 1, 2 demonstrate the BMSTVI model,


(1) $$\begin{array}{l} \log\left(y_{it}\right)=\psi_{t}+\omega_{u}+\delta_{s}+\phi_{p}+\gamma_{c}+\xi_{i}+\varepsilon_{it} \end{array}$$

(2) $$\begin{array}{l} \psi \sim N_{RW}\left(0,{\left[\tau_{\psi }R_{t}\right]}^{-}\right),\;\omega \sim N\left(0,\sigma_{\varepsilon }^{2}\right),\;\delta \sim N\left(0,\sigma_{\varepsilon }^{2}\right),\\ \phi \sim N\left(0,\sigma_{\varepsilon }^{2}\right),\;\gamma \sim N\left(0,\sigma_{\varepsilon }^{2}\right),\;\xi \sim N_{iCAR}\left(0,{\left[\tau_{\xi }R_{s}\right]}^{-}\right),\\ \varepsilon_{it}\sim N\left(0,\sigma_{\varepsilon }^{2}\right) \end{array}$$

where *y*_*it*_ represents the MMR value for county *i* at time *t*; ψ_*t*_ is the temporal intercepts (*TIs*) of MMR, representing the overall temporal trend at the national level; ω_*u*_ is the spatial intercepts (*SIs*) of MMR at the rural–urban differences, representing the spatial pattern of MMR within this administrative division, further *u* denotes urban or rural areas; δ_*s*_ is the *SIs* of MMR across the seven major geographical regions in China; ϕ_*p*_ is the *SIs* at the provincial level; γ_*c*_ is the *SIs* at the city level; ξ_*i*_ is the *SIs* at the county level; and ε_*it*_ represents the spatiotemporal residuals of the model. ψ_*t*_ follows the random walk (RW) prior model, ξ_*i*_ follows the intrinsic conditional autoregressive (iCAR) prior model, and all the other five components follow the independent and identically distributed prior model.

Second, building on the outputs of the BMSTVI model, we quantified the spatiotemporal contributions of five administrative scales using the STVPI, further refining the policy implications (38). The STVPI method offers flexibility by enabling adjustments in the combination of random effects and accommodating additional evaluation dimensions, thus allowing for a more nuanced understanding of spatiotemporal scale impacts. In this study, the STVPI provides the relative explanatory percentages for each administrative scale, ensuring cross-scale comparability through its unified, full-map modeling framework and standardized data structure. A higher STVPI value indicates a greater relative influence of the corresponding administrative scale on MMR disparities. Moreover, Bayesian credible intervals (2.5%−97.5%, 25%−75%) for STVPI values were obtained through joint posterior sampling of hyperparameters in random effects components, facilitating assessment of statistical uncertainty in our findings.

## 3 Results

### 3.1 Spatiotemporal inequity in maternal mortality in china

**Figure 3:**
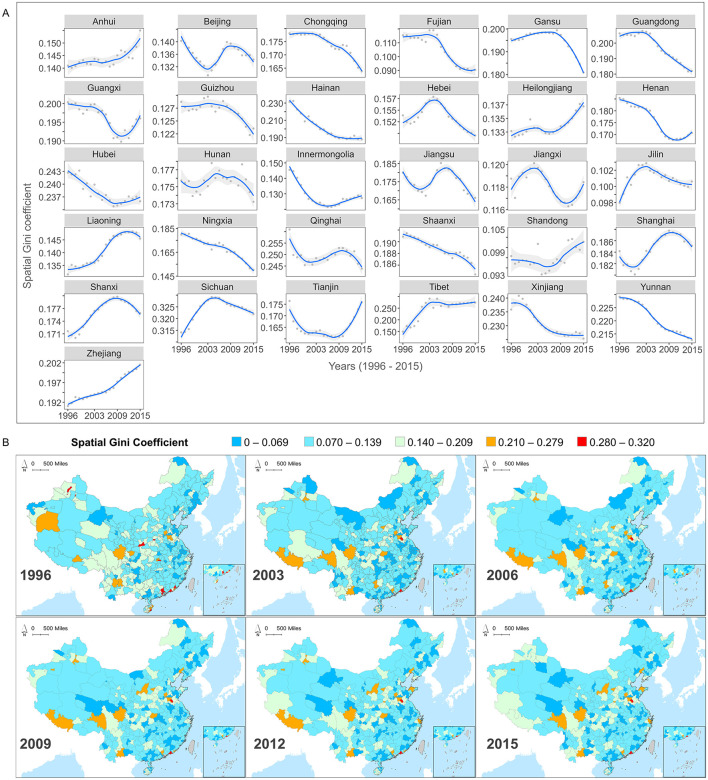
**(A)** Temporal inequity trends of maternal mortality ratios (MMR) among provinces from 1996 to 2015. **(B)** The maps showing spatial inequity of MMR within cities (1996, 2003, 2006, 2009, 2012, 2015).

Using county-level MMR panel data, we constructed temporal trend graphs of the spatial Gini coefficients for each province in China from 1996 to 2015 (Figure 3A) and visualized the distribution of these coefficients at the city scale (Figure 3B). Overall, the highest spatial Gini coefficients for MMR at both the provincial and city levels remained below 0.33, indicating a relatively equitable spatial distribution of MMR across provinces and urban areas from 1996 to 2015.

From a temporal perspective (Figure 3A), the spatial Gini coefficients for different provinces exhibited diverse fluctuating trends throughout the study period. For instance, the spatial Gini coefficients are relatively high in southeastern provinces (e.g., Fujian and Guangdong), and in southwestern provinces (e.g., Yunnan). Showed a consistent linear decline, indicating that MMR inequity within these provinces has gradually improved. However, most provinces exhibited non-linear trends in spatial Gini coefficients. Provinces in northern China (e.g., Beijing) and in the eastern coastal area (e.g., Jiangsu and Shanghai). Experienced fluctuations characterized by decreases followed by increases and then further decreases, indicating unstable internal MMR inequities. In contrast, provinces such as Heilongjiang, Guangxi, and Jiangxi, displayed initial decreases followed by increases, suggesting unresolved issues in internal inequities that require ongoing monitoring of MMR variations among counties and timely policy adjustments. Furthermore, while provinces such as Zhejiang and Anhui maintained relatively low MMR levels over time, their spatial Gini coefficients steadily increased, indicating increased inequities in MMR among counties within these provinces. Investigating the underlying causes and implementing targeted policies are essential.

In addition to the 31 provincial levels, we calculated spatial Gini coefficients at the scale of 360 cities to examine MMR spatial inequities at finer scales (Figure 3B). We divided it into equitable intervals and cities with colors closer to blue represent better spatial equity while those closer to red indicate higher levels of spatial inequity. Results showed that although MMR had gradually decreased in cities over the study period, the spatial inequity pattern between regions remained relatively stable, forming a “Spatial Inequity Lock-In” (SILI) effect. For example, regions like Aba Tibetan and Qiang Autonomous Prefecture, Liaocheng, and Jining, maintained high values, indicating persistent MMR inequity issues that were not alleviated over the 20-year study period. In contrast, low-value areas such as the Greater Khingan region, Jilin, and Baishan, exhibited consistently low levels of inequity. Notably, while MMR in the Tibet Autonomous Region, Guangxi Zhuang Autonomous Region, and Sichuan Province declined at the provincial level, city-level spatial inequities increased, revealing increased MMR disparities in these regions. The persistent pattern of unbalanced spatial distribution throughout the study period underscores a process where the cumulative advantages and disadvantages in the allocation of healthcare resources and the outcomes of improvements have become progressively entrenched. This observation provides further evidence of the “Matthew effect” in the field of maternal health, where inequities are not only sustained but increasingly solidified over time.

Overall, the MMR spatial Gini coefficient reveals the significant regional relative inequity that still exists in China's efforts to reduce MMR, particularly evident at the smaller administrative levels. Additionally, the long-term SILI effect further demonstrates that even with the nationwide implementation of unified MMR reduction policies, there remains notable disparities in the actual outcomes across different regions.

### 3.2 Spatiotemporal cluster patterns of MMR in china

**Figure 4:**
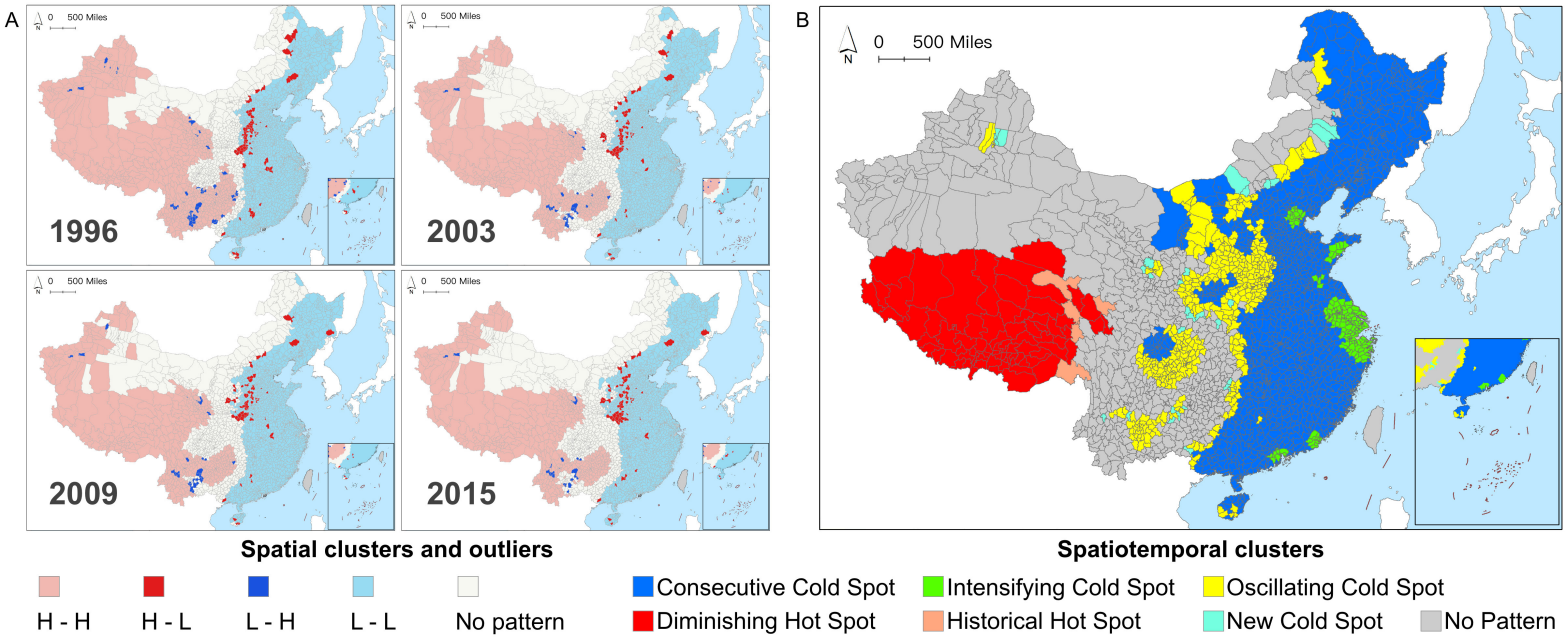
**(A)** Spatial clusters and outliers of Chinese county-level maternal mortality ratios (MMR) in 1996, 2003, 2009, and 2015. **(B)** Spatiotemporal hot spot patterns of Chinese county-level MMR during 1996–2015.

Figure 4 illustrates the spatiotemporal geographic cluster patterns of MMR at the county level in China. Specifically, Figure 4A reveals the geographic cluster and outlier distribution patterns of county-level MMR in four specific years (1996, 2003, 2009, and 2015), while Figure 4B presents the results of the county-level MMR spatiotemporal cold and hot spot analysis.

Specifically, the MMR of counties in China exhibited a distinct east-west heterogeneity: western regions were predominantly characterized by high-high clusters (indicating concentrated spatial distribution of counties with high MMR), whereas eastern regions showed low-low clusters. Outliers were primarily located at the intersections of clustering and non-detection patterns, indicating that the MMR of these counties significantly differed from neighboring counties. With the national decline in MMR, two main spatial patterns of outliers emerged: the Henan-Hebei region demonstrated high-MMR counties surrounded by low-MMR counties, suggesting that, despite achieving or nearing the SDGs target, substantial inequities in maternal health persisted compared to adjacent areas. Conversely, the Yunnan-Guizhou region exhibited low-MMR counties surrounded by high-MMR counties, indicating relatively remarkable progress in reducing MMR. Counties with undetected patterns were mainly distributed in northern and central interstitial regions, where MMR did not show statistically significant clustering or outlier characteristics. From a longitudinal perspective, the spatial clustering and outlier patterns across different years showed high similarity, further verifying the presence of the “spatial lock-in effect”. This effect indicated that despite China's substantial progress in reducing the overall MMR during the study period, the patterns of clustering and anomalies in its spatial distribution remained largely consistent. Results suggested that spatial inequities were both persistent and enduring.

As Figure 4A only captured the spatial clustering and outlier patterns of MMR across regions, lacking a longitudinal perspective, we further employed emerging hot spot analysis. This method helped identify spatiotemporal MMR hot and cold spots across Chinese counties from 1996 to 2015 (Figure 4B). Spatiotemporal hot spots represented sustained high-value MMR clustering across adjacent time steps (1 year), while cold spots indicated persistent low-value clustering. Results revealed six spatial-temporal clustering patterns among 2,894 counties. Given that MMR gradually declined across most regions in the study period, cold spot counties (2,294, 79.27%) far outnumbered hot spot counties (50, 2.76%). Low-MMR regions were concentrated in the east, while high-MMR areas were mostly in the southwest. Non-detected counties (520, 17.97%) represented areas where MMR spatiotemporal clustering patterns did not reach statistical significance.

Specifically, hotspot counties were mainly concentrated in the Tibet Autonomous Region and Qinghai Province, with 75 counties (2.59%) identified as hotspot-decreasing counties. While the MMR in these counties remained relatively high over time, it gradually declined, though their progress was slower compared to surrounding areas. Additionally, five counties (0.17%) were recognized as historical hotspots, indicating notable reductions in MMR and their gradual departure from high-value clusters, making them successful examples for neighboring regions, particularly those with similar natural and socio-economic conditions.

Besides, cold spot counties were further divided into four categories: Consecutive Cold Spot, New Cold Spot, Intensifying Cold Spot, and Oscillating Cold Spot. Consecutive cold spots (1,598, 55.22%) were concentrated in the eastern regions and parts of Sichuan, Inner Mongolia, and Shaanxi provinces, indicating consistently low and stable MMR levels throughout the study period. New cold spots (31, 1.07%) were mostly in Inner Mongolia and Guizhou provinces, scattered in Xinjiang Uygur Autonomous Region, Gansu, and Shaanxi provinces, signifying counties achieving noteworthy MMR reduction for the first time, with further support required to consolidate these gains. Intensifying cold spots (214, 7.39%) formed clusters around Zhejiang, Tianjin, Shanghai, and Guangdong, indicating counties with progressively greater MMR reductions than neighboring counties. The oscillating cold spots (451 counties, 15.58%) were primarily concentrated in Central China, followed by Inner Mongolia, Yunnan, Sichuan, and sporadically distributed in certain areas of Xinjiang and Hainan. The decline in MMR within these counties exhibited instability, highlighting the necessity for dynamic policy adjustments and sustained long-term monitoring. In general, spatiotemporal hot spot analysis revealed diverse spatiotemporal trends in county-level MMR in China, underscoring the need for more flexible and diverse policies to further reduce MMR and address inter-regional inequities.

### 3.3 Administrative scale impacts under spatiotemporal coupling

**Figure 5:**
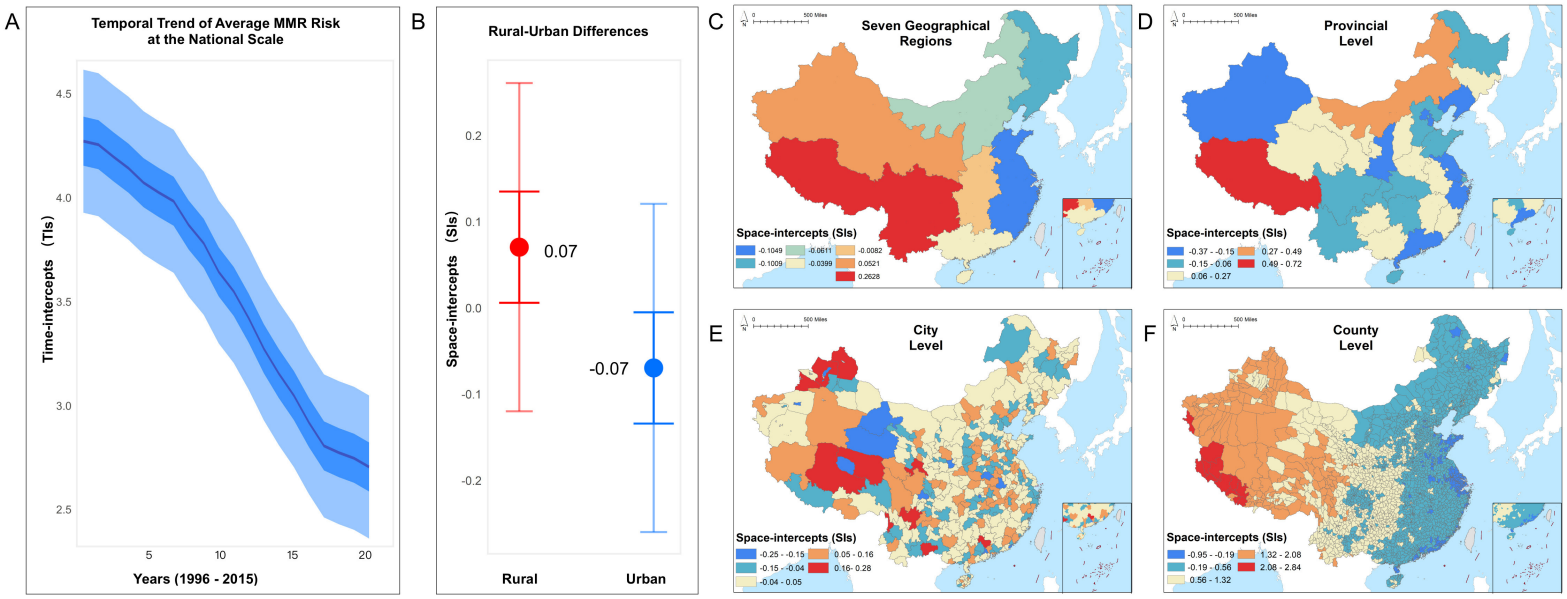
The relative average risks of maternal mortality ratio (MMR), estimated by the BMSTVI model, encompassing the temporal trend and multiscale spatial distribution, with panels showing **(A)** national scale trend, **(B)** rural–urban differences, **(C)** seven geographical regions, **(D)** provincial level, **(E)** city level, and **(F)** county level.

From the perspective of spatiotemporal coupling, we further explored potential causes of spatiotemporal inequity and spatiotemporal clusters that influenced county-level MMR. First, from a temporal perspective, we applied a BMSTVI model to map the time intercept (TIs) curves for MMR in China from 1996 to 2015, displaying uncertainty with wide (95%) and narrow (50%) confidence intervals (Figure 5A). Results indicated a general declining trend in MMR over the past two decades. Although the decline rate was relatively slow from 1996 to 2003 and from 2012 to 2015, MMR still decreased steadily, with an accelerated rate of decline observed between 2003 and 2012, which was likely to associate with two major healthcare reforms during this period.

Second, in the spatial dimension, we examined the relative average risk of MMR across five administrative levels. Results show that MMR risk is higher in rural areas compared to urban areas (Figure 5B). To gain insights into MMR risks at smaller administrative levels, we analyzed average MMR risk across seven geographic divisions (Figure 5C), 31 provinces (Figure 5D), 360 cities (Figure 5E), and 2,894 counties (Figure 5F). The risk was mapped in equal intervals within each scale, with darker colors indicating higher average risk in that region.

Overall, MMR risk is generally higher in the western regions than that in the eastern areas. Among the geographic divisions, lower MMR risk areas are primarily concentrated along the eastern coast. The analysis shows that southwestern and northwestern regions exhibit the highest MMR risks. At the provincial level, Tibet in the southwest, as well as Qinghai and Gansu in the northwest, display relatively higher risk, as indicated by the darker shading. At the city level, Tibet's Ngari and Nagqu Prefectures, Qinghai's Yushu Tibetan Autonomous Prefecture and Golog Tibetan Autonomous Prefecture, and Gansu's Gannan Tibetan Autonomous Prefecture and Wuwei city exhibit elevated MMR risks compared to other cities in the same provinces. At the county level, Zhada and Geji Counties in Ngari, Nima and Shenza Counties in Nagqu, Qumarleb and Zadoi Counties in Yushu, and certain counties within Golog and Gannan Prefectures, as well as Wuwei city, have higher MMR risks compared to other counties within the same city. Counties in the central and northern-central regions show lighter shading, indicating relatively lower MMR risk, while the eastern regions have the lightest shading, indicating the lowest MMR risk. The spatial distribution of county-level data reveals a distinct increasing pattern of MMR risk from east to west in China.

Although previous analysis has identified the spatiotemporal differences in MMR, the implementation of effective interventions requires identifying appropriate administrative intervention scales within sensitive areas to maximize the impact of MMR reduction efforts. We therefore calculated the STVPI and its uncertainty (wide and narrow credible intervals) to reveal the relative importance of each administrative scale in contributing to spatiotemporal differences in MMR (percentage of contribution).

**Figure 6:**
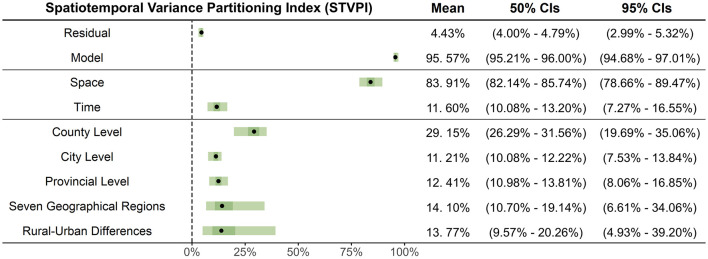
The relative contributions of different components of the BMSTVI model using STVPI, including: model and residual, temporal and spatial effects, and five spatial administrative levels (rural–urban differences, seven geographical regions, provincial level, city level, and county level).

Figure 6A shows that the STVPI model explains 95.57% of the total variance (95% Credible Intervals (CIs): 94.68%−97.01%), with a residual variance contribution (ρ_*residual*_) of 4.43% (95% CIs: 2.99%−5.32%), indicating is a good reliability of the model. The temporal and spatial effects (Figure 6B) contribute 11.60% (95% CIs: 7.27%−16.55%) and 83.91% (95% CIs: 78.66%−89.47%) to MMR, respectively. This result suggests that a nationwide, one-size-fits-all policy focusing solely on temporal adjustments has a limited effect impact on MMR spatiotemporal variation, emphasizing the necessity for context-specific policy interventions tailored to local conditions. Spatially, county-level analysis emerged as the most crucial scale for interventions, accounting for 29.15% (95% CIs: 19.69%−35.06%) of the spatiotemporal variability in MMR, followed by regional analysis across the seven geographic divisions, which could address an additional 14.10% (95% CIs: 6.61%−34.06%) of the variability. Rural–urban policies contribute to 13.77% (95% CIs: 4.93%−39.2%) of MMR variability, followed by provincial-level policies at 12.41% (95% CIs: 8.06%−16.85%) and city-level interventions, which account for 11.21% (95% CIs: 7.53%−13.84%) (Figure 6C). Altogether, these five administrative levels account for over 80% of the spatiotemporal variance in maternal mortality, indicating that a multilayered, spatially targeted approach, primarily led at the county level, would yield greater practical efficacy in reducing MMR.

## 4 Discussion

Coordinated and sustained efforts are essential to address persistent spatiotemporal disparities in maternal mortality (39), especially small-area inequities in developing countries like China (18). Previous studies have often overlooked the complex interplay among spatial, temporal, and administrative factors, limiting the understanding of these disparities (27). To fill this gap, this study analyzed 20 years of county-level maternal mortality data in China to uncover small-area spatiotemporal disparities and assess their dependence on administrative scale effects. From these two-perspective spatiotemporal assessments, this study provided the following interrelated contributions. First, we identified and visualized persistent spatiotemporal inequities in MMR at the small area level by employing advanced spatial analysis techniques, including spatial Gini coefficients and cluster detection methods. We uncovered a novel phenomenon termed the “SILI” effect, which highlighted the temporally varying but spatially stable inequity patterns over China, despite considerable national-level improvements. Second, by applying the BMSTVI model and STVPI, we quantified the contributions of five administrative levels to maternal mortality disparities, highlighting the county level as the most critical for intervention and providing actionable insights for region-specific, time-sensitive health policies. This research provides novel evidence and a layered framework integrating spatiotemporal disparities with their underlying drivers, laying the foundation for targeted maternal health interventions.

First, although China has significantly reduced national maternal mortality rates through programs such as Reducing Maternal Mortality and Eliminating Neonatal Tetanus, improved healthcare services and education, increased numbers of midwives, and enhanced socioeconomic conditions (28), the spatial Gini coefficient of county-level MMR distribution remained largely unchanged, indicating persistent spatial inequities over time. Moreover, these persistent inequities were accompanied by sustained spatiotemporal clustering patterns. These findings sharply contrast with prior research suggesting improvements in maternal mortality equity across China (40). In this study, we termed this phenomenon the “SILI effect,” highlighting that county-level MMR disparities exhibited persistent, stable spatial inequity patterns that failed to diminish in line with national progress over time. The persistence of the SILI effect underscores significant challenges for further reducing small-area maternal mortality in China.

One potential explanation for this SILI phenomenon was the concentration of healthcare resources in economically developed and highly accessible regions, resulting in more effective MMR reduction there, while less-developed areas struggled to allocate and utilize their limited healthcare resources efficiently (31, 41–43). Another contributing factor was regional inconsistency in health development strategies, hindering balanced progress toward reducing MMR (25). Additionally, a diminishing “catch-up effect” indicated that regions with initially higher MMR achieved reductions faster than regions with initially lower MMR, narrowing the gap unevenly. Furthermore, regional differences in customs, educational backgrounds, and policy implementation may exacerbate imbalances in MMR reduction. Future interventions should comprehensively address these influencing factors by optimizing healthcare resource allocation mechanisms (44–46), formulating targeted development policies, and strengthening maternal health education to promote equitable achievement of sustainable maternal health goals in small areas. Particular attention should be given to regions exhibiting persistent inequities or identified as spatial outliers to better understand the underlying drivers of their elevated MMR and to implement evidence-based, tailored interventions.

Second, from a spatiotemporal clustering perspective, we conducted spatial clustering and outlier analyses to reveal geographic cluster patterns of MMR at the county level in China, highlighting the occurrence of high-low and low-high outliers. The high-low outliers might be explained by the “spatial siphon effect,” where healthcare resources disproportionately flow from underdeveloped regions to more developed areas. This imbalance in resource allocation enables more developed regions to achieve greater reductions in MMR (47). Addressing this issue requires targeted strategies such as promoting local economic development, enhancing healthcare infrastructure, retaining and attracting medical talent, and establishing reciprocal medical support systems. Conversely, low-high outliers identified in regions like Sichuan and Chongqing indicate that improvements in healthcare resources within certain counties can positively impact neighboring areas through a “spatial spillover effect,” thereby driving further MMR reductions across these regions. However, the effectiveness of this spillover process may be influenced by factors such as maternal awareness of prenatal care, adequacy of healthcare resources, and transportation accessibility (36, 37, 48, 49). These findings suggest that governments should comprehensively consider both intra- and inter-regional interactions when designing tailored policies aimed at further reducing small-area MMR.

Third, from a spatiotemporal coupling perspective, we employed emerging spatiotemporal hotspot analysis to illustrate the dynamic evolution of MMR over time, identifying cold and hot spot patterns in small-area MMR and locating priority regions (hotspot counties). For counties with persistently low and relatively equitable MMR, categorized as cold spots, existing policies should continue. For high-priority intervention areas, we identified two types: hotspot counties with consistently high MMR should adopt local-specific measures based on national maternal healthcare strategies, such as increased healthcare investment and implementation of prenatal care services (38, 39, 50, 51), to drive further MMR reduction. Additionally, regions with sustained inequities required specific optimization of current healthcare resource allocation and policies (35), along with targeted investment (52). Policymakers should explicitly consider spatiotemporal interactions when developing and implementing interventions to effectively reduce MMR and address intra- and inter-regional disparities, thereby avoiding a “one-size-fits-all” approach.

Fourth, our study revealed trends in average MMR under temporal effects and across various administrative levels (spatial effects). The BMSTVI model indicated a sharp decline in average MMR across Chinese counties from 2003 to 2012, which was consistent with previous studies (53). This finding was likely associated with the synergistic effects of two national policies. The first was the universal “Reduction of Maternal Mortality and Elimination of Neonatal Tetanus” policy. The second was the “Health Reform” policy, which aimed to establish basic health insurance and enhance maternal and child health services (54). Further, by clarifying the distribution of maternal mortality risk within five administrative divisions, our results emphasized the importance of multi-layered analysis that spanned from broad macro-scale geographic contexts to specific micro-scale county-level perspectives. Findings indicated that the highest MMR was observed in southwest China, aligning with previous studies (18, 37). Apart from this, we introduced a more refined model, which allowed for a progressive identification of specific counties from broader geographic divisions with scientific evidence for policy interventions. This highlighted the necessity of a layered, macro-to-micro policy intervention framework, which integrated macro-level (national, rural–urban, and regional policies) for strategic direction, meso-level (provincial and city) for institutional development and resource coordination, and micro-level (county) for direct implementation. By aligning interventions with administrative hierarchies, this structured approach ensured that national maternal health (MMR) goals were translated into context-specific interventions, thereby bridging policy objectives with localized implementation to maximize impact.

Fifth, we used the spatiotemporal variance partitioning index to assess the relative contributions of spatial and temporal effects to better understand the driving mechanisms behind these differences from a spatiotemporal scale perspective. We confirmed that the county level was the optimal intervention scale. We also discovered that spatial effects (83.91%) contributed significantly more to MMR variance than temporal effects (11.60%), possibly due to pronounced differences in healthcare resources, service levels, and socioeconomic status across regions, which amplified spatial disparities in MMR (45). Consequently, spatially targeted intervention measures were crucial. Last, to maximize the spatial intervention efficacy of MMR, we quantified the relative significance of each administrative scale to MMR. Results showed that rural–urban dichotomous policies addressed 13.77% of MMR spatiotemporal variability, policies targeting seven geographic regions addressed 14.10%, provincial-level policies explained 12.41%, city-level policies contributed to a further 11.21%, and policies at the smallest scale, the county level, had an influence of 29.15%.

The results highlighted the critical role of county-level policy interventions in reducing MMR, while policies at other administrative levels also remain essential. Strengthening national-level design is crucial to address rural–urban and regional disparities through financial support and optimized healthcare resource allocation, ensuring equitable access to maternal health services (55–57). Provincial and city governments should adapt national policies to local contexts, redistribute medical resources to underserved areas, optimize healthcare infrastructure, and ensure precise implementation (58). County governments, as the primary policy executors, should focus on enhancing primary healthcare systems, service accessibility, and maximizing intervention effectiveness (59–61). This multi-tiered governance framework integrates top-down enforcement with bottom-up adaptability, fostering scalable, context-sensitive solutions through cross-agency coordination. Given the global presence of similar administrative structures and scale effects, the analytical framework proposed here can serve as a valuable reference for improving maternal health governance in other countries.

Finally, from a policy perspective, our findings presented a comprehensive macro-to-micro analytical framework for global MMR analysis and provided valuable insights for designing multilevel, region-specific intervention strategies. This multi-level analysis not only systematically revealed significant spatiotemporal inequities between regions but also offered scientific evidence for accurately identifying priority intervention areas, thus providing solid theoretical support for designing region-specific strategies from broad to localized levels. Our research serves as a reference for global MMR interventions. At the macro level, addressing the SILI effect requires governments to tackle deep-rooted maternal health disparities by increasing medical investment in disadvantaged regions and optimizing the spatial distribution of existing health resources. Additionally, broader economic development and coordinated health economic strategies are needed to resolve regional health resource imbalances (62, 63) caused by unequitable investment and allocation, which contribute to entrenched health inequity (45, 46). At the micro level, addressing noteworthy spatiotemporal MMR differences requires considering the unique ecological environments and resource conditions of small areas (64), focusing on disadvantaged counties, and promoting the successful experiences of demonstration counties to guide surrounding areas. Furthermore, based on the influence of each administrative scale on MMR, selecting the appropriate intervention scale would help countries more efficiently achieve SDG 3.

Our study has several limitations. First, due to monitoring methods and resource limitations, we only collected MMR monitoring data from 1996 to 2015, which may not fully reflect recent progress in addressing small-area MMR disparities. Fortunately, our findings still discovered significant spatiotemporal heterogeneity and SILI effect, which could provide valuable reference for current intervention policies. Second, this study was a macro-ecological study and may contain ecological fallacies and uncontrolled confounding factors. However, our study employed data with broad, fine-scale, and geographically comprehensive population coverage, which would offer insights to sustainably reduce MMR. Additionally, China's case reporting system, particularly in remote or rural areas, may lead to underreporting or delayed reporting of maternal deaths. Finally, social and environmental determinants, such as socioeconomic status, education level, air pollution, and climate change, may contribute to maternal health inequities, we would fully consider these factors in the future research (18). We recommend that future research explores observable factors beyond administrative boundaries, such as social and environmental determinants, and applies more advanced spatiotemporal statistical methods to achieve greater precision (36), thereby providing robust evidence for designing targeted intervention policies.

## 5 Conclusions

This study provided a comprehensive analysis of the spatiotemporal variation in maternal mortality across Chinese counties from 1996 to 2015, revealing three key findings. First, from a macro perspective, the proportion of counties meeting the SDG 3 maternal health indicator increased from 27.05% to 93.40%, yet persistent spatiotemporal inequities and stable clustering patterns highlighted the Spatial Inequity Lock-in Effect and long-standing regional disparities in maternal health. This underscored the ineffectiveness of a universal, one-size-fits-all approach to addressing MMR. Second, at the meso-level, spatial dimension accounted for 83.91% of MMR variation, far surpassing the temporal dimension (11.60%), with the county level being the most influential (29.15%). These findings emphasized the importance of county-level policies in reducing MMR. Finally, micro-level spatiotemporal hotspot analysis identified hotspot and coldspot counties, highlighting priority areas for intervention and successful examples. This targeted approach underscored the need for micro-level analysis to pinpoint high-risk areas and optimize intervention strategies. In summary, our study proposed a multi-level spatiotemporal evaluation framework, integrating macro, meso, and micro perspectives to assess MMR within the SDGs framework. This framework provides a valuable reference for formulating context-specific, dynamic interventions to address maternal health inequities and advance SDG targets.

## Data Availability

The raw data supporting the conclusions of this article will be made available by the authors, without undue reservation.
